# Circulating inflammation signature predicts overall survival and relapse-free survival in metastatic colorectal cancer

**DOI:** 10.1038/s41416-018-0360-y

**Published:** 2019-01-14

**Authors:** Andreas Varkaris, Anastasia Katsiampoura, Jennifer S. Davis, Neeraj Shah, Michael Lam, Rosa Lizeth Frias, Cristina Ivan, Masayoshi Shimizu, Jeffrey Morris, David Menter, Michael Overman, Hai Tran, John Heymach, Yun Shin Chun, Jean-Nicolas Vauthey, George Calin, Scott Kopetz

**Affiliations:** 1 Department of Hematology Oncology, Beth Israel Deaconess Medical Center, Harvard Medical School, Boston, MA USA; 2 Department of Gastrointestinal Medical Oncology, Division of Cancer Medicine, The University of Texas MD Anderson Cancer Center, Houston, TX USA; 3 St. Elizabeth’s Medical Center, Boston, MA USA; 4 Department of Epidemiology, Division of Cancer Prevention and Population Sciences, The University of Texas MD Anderson Cancer Center, Houston, TX USA; 5 Department of Experimental Therapeutics, Division of Basic Science Research, The University of Texas MD Anderson Cancer Center, Houston, TX USA; 6 Department of Biostatistics, Division of Science, The University of Texas MD Anderson Cancer Center, Houston, TX USA; 7 Department of Thoracic/Head and Neck Medical Oncology - Research, Division of Cancer Medicine, The University of Texas MD Anderson Cancer Center, Houston, TX USA; 8 Department of Thoracic/Head and Neck Medical Oncology, Division of Cancer Medicine, The University of Texas MD Anderson Cancer Center, Houston, TX USA; 9 Hepato-Pancreato-Biliary Section, Department of Surgical Oncology, The University of Texas MD Anderson Cancer Center, Houston, TX USA

**Keywords:** Colorectal cancer, Tumour biomarkers

## Abstract

**Background:**

Metastatic colorectal cancer (mCRC) is a highly heterogeneous disease from a clinical, molecular, and immunological perspective. Current predictive models rely primarily in tissue based genetic analysis, which not always correlate with inflammatory response. Here we evaluated the role of a circulating inflammatory signature as a prognostic marker in mCRC.

**Methods:**

Two hundred eleven newly diagnosed patients with mCRC were enrolled in the study. One hundred twenty-one patients had unresectable metastases, whereas ninety patients had potentially resectable liver metastases at presentation. Analysis of miR-21, IL-6, and IL-8 in the plasma of peripheral blood was performed at baseline. Patients with high circulating levels of ≥2 of the three inflammation markers (miR-21, IL-6, and IL-8) were considered to have the “Inflammation phenotype-positive CISIG”.

**Results:**

Positive CISIG was found in 39/90 (43%) and 50/121 (45%) patients in the resectable and unresectable cohort, respectively. In the resectable population the median relapse-free survival was 18.4 compared to 31.4 months (*p* = 0.001 HR 2.09, 95% CI 1.2–3.67) for positive vs. negative CISIG. In contrast, the individual components were not significant. In the same population the median overall survival was 46.2 compared to 66.0 months (*p* = 0.0003, HR 2.57, 95% CI 1.26–5.27) for positive vs. negative CISIG, but not significant for the individual components. In the unresectable population, the median overall survival was 13.5 compared to 25.0 months (*p* = 0.0008, HR 2.49, 95% CI 1.46–4.22) for positive vs. negative CISIG. IL-6 was independently prognostic with overall survival of 16.2 compared to 27.0 months (*p* = 0.004, HR 1.96, 95% CI 1.24–3.11) for high vs. low IL-6, but not the other components. Using a Cox regression model, we demonstrated that CISIG is an independent predictive marker of survival in patients with unresectable disease (HR 1.8, 95% CI 1.2, 2.8, *p* < 0.01).

**Conclusion:**

In two different cohorts, we demonstrated that CISIG is a strong prognostic factor of relapse-free and overall survival of patients with mCRC. Based on these data, analysis of circulating inflammatory signaling can be complimentary to traditional molecular testing.

## Background

Better understanding of colorectal cancer (CRC) biology led to the development of novel combination therapies that prolong patients’ survival.^1^ Nevertheless, approximately a quarter of patients with stage II and the majority of patients with stage III and IV disease require multiple lines of systemic therapy and eventually succumb from the disease.^2^ Identification of patients with increased risk of progression is critical to understand critical components of the disease biology, prioritize therapeutic interventions, and improve clinical outcomes.

The association of chronic inflammation and CRC tumorigenesis is well established and in the last decade has received growing supportive evidence from epidemiological, genetic, and pharmacological studies. The crosstalk between inflammatory, stromal, and cancer cells occurs in multiple levels allowing the tumor cells to undergo genetic, epigenetic, and biochemical changes to acquire an aggressive phenotype. MicroRNAs are a critical component of this cycle. miR-21 is specifically relevant to CRC tumorigenesis.^3,4^ Multiple studies have demonstrated that miR-21 is overexpressed in CRC human specimens, whereas the level of expression is higher in progressive stages of the disease.^3,5–8^ Animal studies demonstrated that miR-21 is associated with inflammation-induced CRC tumorigenesis.^9,10^ Using human samples, Schetter et al. demonstrated that the pro-inflammatory effect of miR-21 is mediated through cytokine upregulation including IL-6 and IL-8, two cytokines that have also been directly associated with CRC progression.^11^ Aberrant expression of miR-21 in tumor tissue is associated with reduced disease-free survival (DFS) and poor overall survival (OS) of patients with CRC, independently of clinical pathologic and major tumor genetic features.^8,12,13^ Further, Sarlinova et al. demonstrated that mirR-21 expression is significantly increased in the peripheral blood of patients with CRC, compared to matched healthy individuals.^14^ Based on this knowledge, three independent studies evaluated the role of serum miR-21 levels as a biomarker in CRC. In contrary to studies performed on tissue samples, serum levels of miR-21 alone have limited prognostic value.^15^

Tissue-based analyses have not always well reflected the current state of the tumor-host interactions. Indeed, cancer cachexia, systemic inflammatory response, and tumor-associated symptom burden are poorly evaluated by studies of the tumor tissue. Additionally, tumor-based assessments are limited to a single time-point in the disease course and do not fully reflect the current state of the disease. Circulating markers, in contrast, provide temporal relevance and integrate a systemic response to the tumor.

On the basis of this rationale, in this study, we examined the role of Circulating Inflammation SIGnature (CISIG) determined by high miR-21, IL-6, and IL-8 circulating levels as a potential marker in metastatic CRC. In two different cohorts, we demonstrated that CISIG is a strong prognostic factor of progression-free and overall survival of patients with metastatic CRC. Therefore, the inflammatory signature might be valuable tool in secondary prevention and patient selection for aggressive medical management.

## Methods

### Study cohorts

Two hundred eleven (211) newly diagnosed patients with metastatic colorectal cancer who presented to MD Anderson Cancer Center from April 2002 to December 2008 were enrolled in the study. The patients were divided into two cohorts based on clinical characteristics. The first cohort included one hundred twenty-one (121) patients with unresectable metastases at presentation, whereas the second validation cohort included ninety patients (90) with potentially resectable metastases at presentation. Patients from the second cohort subsequently underwent surgical intervention including partial hepatectomy, with curative intent within 2 years of the date of plasma draw. Patient characteristics are shown in Table 1. The study was carried out with the approval of MD Anderson Cancer Center Institutional Review Board, in accordance to the declaration of Helsinki.

**Table 1:** Patient characteristics

Characteristic	Unresectable cohort		Resectable cohort		*p*-value
Sex	No.	Freq (%)	No.	Freq (%)	
Female	53	43.8	36	40	0.67
Male	68	56.2	54	60	
Race					
White	92	76.1	70	77.8	0.26
Black	16	13.2	6	6.7	
Hispanic	8	6.6	11	12.2	
Other	5	4.1	3	3.3	
Age (median-y)	56		57		0.23
# Metastatic sites					
1	54	44.6	79	87.8	<0.001
2	43	35.5	8	8.9	
≥3	24	19.9	3	3.3	
Prior surgery					
Yes	66	54.5	59	65.5	0.12
No	55	45.5	31	34.5	
Site					
Rectum and sigmoid	62	51.2	48	53.3	0.49
Descending colon	9	7.5	8	9	
Transverse colon	5	4.1	9	10	
Ascending colon	18	14.9	11	12.2	
Cecum	24	19.8	12	13.3	
Unspecified	3	2.5	2	2.2	
Histology					
AdenoCA	104	85.9	75	83.3	0.81
Mucinous adeno CA	15	12.4	14	15.6	
Adenosquamous CA	2	1.7	1	1.1	
Inflammation phenotype					
Yes	52	43.7	40	44.4	1.00
No	67	56.3	50	55.6	

### Blood collection

Human plasma samples for CISIG analysis were obtained after informed consent had been obtained from the patient, per Institutional Review Board (IRB) protocol at the MD Anderson Cancer Center (MDACC). Samples were drawn at the time of presentation, and before initiation of any systematic therapy.

### Patients follow-up

Progression-free survival (PFS) was defined as the interval between enrollment on the study and clinical, laboratory or radiologic progression, or death from any cause. Relapse-free survival (RFS) was defined as the interval between liver resection and recurrence in liver or elsewhere, or death from any cause. Overall survival (OS) was defined as the interval between enrollment on the study and death from any cause.

### Sample collection and storage

Plasma samples were drawn and collected in ethylenediaminetetraacetic acid tubes and centrifuged at 2800 r.p.m. for 10 min at −22 °C. Sample aliquots were placed into 0.5-mL cryovials and stored at −70 °C to −80 °C until analysis. Plasma samples (in 500-μL aliquots) were thawed in parallel and each used for suspension bead multiplex assays.

### MicroRNA isolation and quantitative RT-PCR

For microRNA-based RT-PCR assays, 2.5 µL of enriched small RNAs from plasma samples were reverse transcribed using the TaqMan MicroRNA Reverse Transcription Kit (Applied Biosystems, San Diego, CA) according to manufacturer’s instructions in a total reaction volume of 7.5 µL. A 1∶20 dilution of RT products was used as template for the PCR stage. PCR reaction was performed in triplicate wells using TaqMan 2× Universal PCR Master Mix with conditions as described previously. No-template controls for both RT step and PCR step were included to ensure target specific amplification. The 7900 Sequence Detection System 2.3 (Applied Biosystems) software defaults were used to compute the relative change in RNA expression by the 2^−ΔΔCt^ method with 95% confidence intervals. Circulating miR-21 levels were determined using levels of mir-16 as a control (normalizer). All hemolytic samples (evaluated by spectrophotometric method) were excluded from the study.

### Cytokine assay

Levels of cytokines and biomarkers in serum were assessed using multiplex bead assay (Bio-Rad Laboratories, Hercules, CA, USA and EMD, Bioscience Research Reagents, Temecula, CA, USA). Briefly, samples were incubated for 1 h on the array plates that were pre-spotted with capture antibodies specific for each protein biomarker. Plates were decanted and washed four times before adding a cocktail of biotinylated detection antibodies to each well. After incubating with detection antibodies, plates were washed four times and incubated with streptavidin-horseradish peroxidase conjugate. All incubations were done for 30 min at room temperature with shaking at 200 rpm. Plates were again washed before adding a chemiluminescent substrate, followed by immediate imaging. Cytokine array data are available upon request to the corresponding author.

### Statistical analysis

Statistical analysis was performed using STATA version 11.0. The cut-off of the highest tertile was used to define high level of circulating miR-21 and the bottom two tertiles were defined as low level, based on previous literature.^3^ For IL-6 and IL-8, the median was used as a cut-off point to dichotomize the levels of these markers to high vs. low. Patients with high circulating levels of >2 of the 3 inflammation markers (miR-21, IL-6, and IL-8) were considered to have the “Inflammation phenotype-positive CISIG”. Spearman correlation coefficients for correlation between circulating miR-21, IL-6, and IL-8 were computed. Median OS, and disease-free survival were estimated non-parametrically using the Kaplan–Meier method and compared by the log-rank test. Cox proportional hazards regression was used to adjust for potential confounders and significant differences were assessed using the log-rank test. Calculations were performed with SPSS-version 23.0 software (IBM Corp., Armonk, NY) and SAS 9.4 (SAS Institute). *p* values of less than 0.05 were considered statistically significant. The OS and corresponding censoring were computed in months from diagnosis to death for each patient. Additionally, progression-free survival and recurrent-free survival was computed for patients and was defined as the interval between blood draw and recurrent disease.

## Results

### Inflammation serum signature is prevalent in metastatic CRC

Circulating miR-21, IL-6, and IL-8 levels were measured at diagnosis of metastatic disease in both resectable and unresectable patient cohorts. Positive circulating inflammation signature (CISIG) was found in 39/90 (43%) and 50/121 (45%) patients in the resectable and unresectable cohort, respectively. The difference in CISIG prevalence between the two cohorts was not statistically significant (*p* > 0.05) (Table 1).

### Correlations of plasma levels of inflammatory markers

The correlation between inflammatory markers was performed using Spearman’s coefficient. In agreement with previous studies performed in CRC tissue samples, the serum levels of miR-21, IL-6, and IL-8 showed statistically significant correlation (*p* < 0.001). In the integrated study population (211 patients), the Spearman’s correlation coefficient between miR-21/ IL-6, miR-21/ IL-8, and IL-6/ IL-8 was 0.38, 0.38, and 0.76 respectively (data not shown).

### Inflammatory signature predicts relapse-free survival and overall survival after hepatectomy

**Fig. 1:**
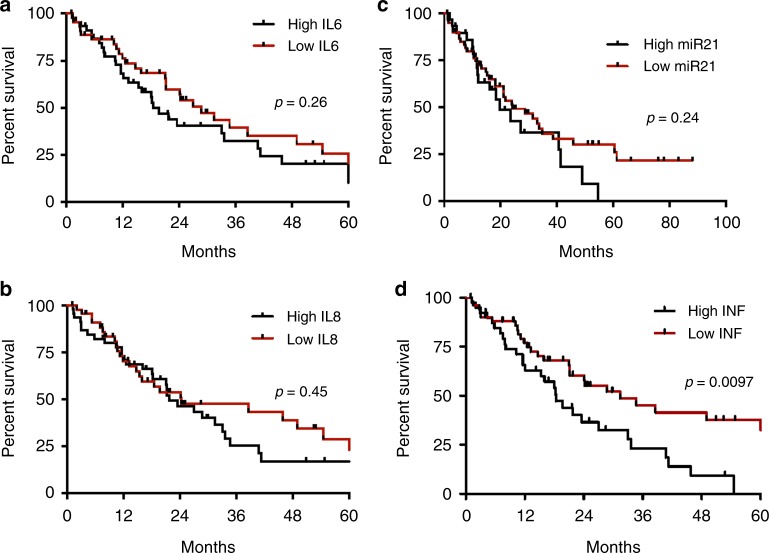
Relapse-free survival after hepatectomy

**Fig. 2:**
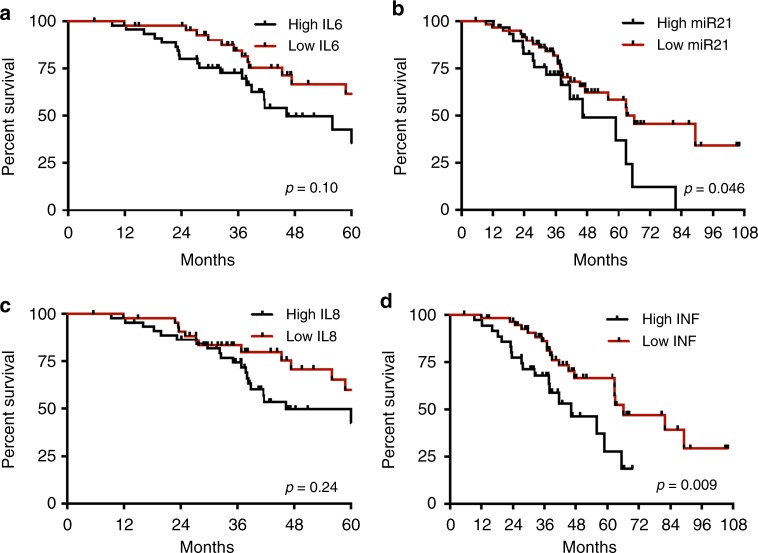
Overall survival in resected cohort

In the resectable population, we evaluated the correlation between inflammatory markers and inflammatory signature at the time of hepatectomy with relapse-free survival, as determined by the time between hepatectomy and relapse of the disease. The median relapse-free survival was 18.4 compared to 31.4 months (*p* = 0.001 HR 2.09, 95% CI 1.2–3.67) for positive vs. negative CISIG. In contrast, the individual components were not significant: 19.7 compared to 24.2 months (*p* = 0.24, HR 1.434, 95% CI 0.79–2.6) for high vs. low miR-21, 18.4 compared to 28.7 months (*p* = 0.26, HR 1.36, 95% CI 0.80–2.31) for high vs. low IL-6, and 21.7 compared to 24.2 months (*p* = 0.61, HR 1.23, 95% CI 0.72–2.80) for high vs. low IL-8, and (Fig. 1 and Supplementary Table 1). In this population, overall survival is further dictated by efficacy of treatment after relapse, and therefore overall survival is also relevant to incorporate risk of recurrence and treatment benefit. The median overall survival was 46.2 compared to 66.0 months (*p* = 0.0003, HR 2.57, 95% CI 1.26–5.27) for positive vs. negative CISIG, but not significant for the individual components: 46.2 compared to 66.0 months (*p* = 0.05, HR 2.1, 95% CI 1.01–4.46) for high vs. low miR-21, 46.2 compared to 66.4 months (*p* = 0.1, HR 1.7, 95% CI 0.90–3.30) for high vs. low IL-6, and 46.2 compared to 62.9 months (*p* = 0.24, HR 1.5, 95% CI 0.77–2.80) for high vs. low IL-8 (Fig. 2; Table 2).

**Table 2:** The prognostic value of circulating markers in metastatic CRC

	Median OS high (m)	Median OS low (m)	HR	95% CI	*p*-value
Resectable cohort					
MiR-21	46.22	66.01	2.124	1.012–4.459	0.046
IL-6	46.20	66.04	1.721	0.896–3.303	0.1
IL-8	46.20	62.95	1.467	0.767–2.802	0.24
Inflammation signature	46.22	66.02	2.572	1.255–5.270	0.09
Unresectable cohort					
MiR-21	19.70	24.20	1.434	0.788–2.611	0.93
IL-6	16.17	27.06	1.961	1.238–3.106	0.004
IL-8	19.10	24.26	0.787	0.148–1.427	0.11
Inflammation signature	13.51	24.98	2.486	1.463–4.223	0.0008

### Inflammatory signature predicts survival in of patients with unresectable disease

**Fig. 3:**
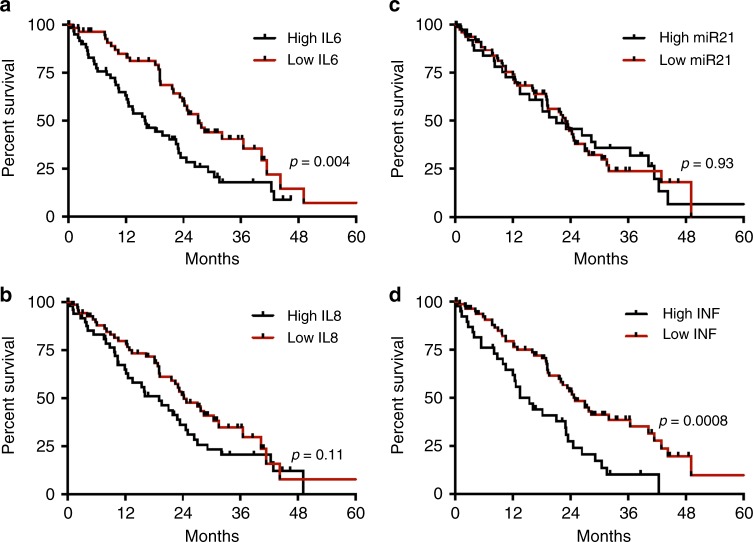
Overall survival in unresected cohort

The role of individual inflammatory markers and the CISIG in predicting survival in patients with unresectable CRC was examined. The median overall survival in this population was 13.5 compared to 25.0 months (*p* = 0.0008, HR 2.49, 95% CI 1.46–4.22) for positive vs. negative CISIG. IL-6 was independently prognostic with overall survival of 16.2 compared to 27.0 months (*p* = 0.004, HR 1.96, 95% CI 1.24–3.11) for high vs. low IL-6, but not the other components: 19.7 compared to 24.2 months (*p* = 0.23, HR 1.43, 95% CI 0.79–2.61) for high vs. low miR-21, and 19.1 compared to 24.3 months (*p* = 0.11, HR 0.8, 95% CI 0.15–1.43) for high vs. low IL-8 (Fig. 3; Table 3).

**Table 3:** Inflammatory signature is an independent predictive marker of survival in patients with unresectable disease

Cox proportional hazards results		
Variable	HR (95% CI)	*p* value
High CISIG (ref = low)	1.8 (1.2, 2.8)	<.01
NLR > 5 (ref = ≤5)	1.4 (0.9, 2.3)	0.15
CEA	1.0 (1.0, 1.0)	0.15
Hemoglobin < 12.4 (ref = ≥12.4)	1.2 (0.8, 1.8)	0.49
Chemotherapy (ref = none)	0.8 (0.6, 1.2)	0.35
Left-sided tumor (ref = right side)	0.6 (0.4, 0.9)	0.02

### Inflammatory signature is an independent predictive marker of survival in patients with unresectable disease

Previous studies have demonstrated that elevated CEA, low hemoglobin, elevated lymphocyte to neutrophil ratio (LNR), elevated WBC, elevated platelets, and previous chemotherapy are associated with poor prognosis in metastatic colorectal cancer.^16,17^ Using our unresected patient cohort, we evaluated the ability of the inflammatory signature to independently predict overall survival. We included variables significantly associated with the inflammatory signature, and known prognostic factors, such as patient sex, and site of the primary tumor. The final model included patient sex, primary tumor site (left vs. right), Hgb < 10 g/dL, CEA, NLR > 5, previous cytotoxic chemotherapy, albumin < 3.5 g/dL, platelets > 310 000 (cells/mcL), and WBC > 8000 (cells/mcL) as covariates. After adjusting for the above covariates, the inflammatory signature was still significantly predictive of overall survival (HR 1.8, 95% CI 1.2, 2.8, *p* < .01). The only other significant predictor in the model was left sided tumor (which was protective, HR: 0.6, 95% CI: 0.4, 0.9, *p* = 0.02) (Table 3). Finally, we examined the correlation of CISIG with other variables used in the model. Our analysis showed that in this patient cohort CISIG was associated with previous chemotherapy, albumin < 3.5, increased CEA, hemoglobin of <12.4, NLR > 5, platelet counts > 310 and WBC > 8. We run these seven factors through model selection for their ability to predict a positive CISIG and found that only albumin < 3.5, hemoglobin < 12.4, platelet counts > 310, and WBC > 8 remained significantly associated with positive CISIG in a multivariate model (Supplementary Table 2).

## Discussion

Individual inflammatory markers and cytokines have previously shown prognostic value in solid tumors in small trials. In this study, we demonstrate that a signature of circulating inflammatory markers correlates with metastatic colorectal cancer aggressiveness. Of note, the prognostic value of the CISIG is evaluated in both patients with resectable and unresectable tumors with similar statistically significant results.

A number of studies demonstrated a correlation between genetic mutations and aggressive disease. The association of Ras mutations with poor disease outcomes after hepatectomy is well documented.^18^ In parallel, the prognostic impact of *BRAF*^V600E^ mutations in metastatic CRC is also dramatic, since patients with *BRAF*^V600E^-mutant CRC survive approximately less than half as long as patients with *BRAF* wild-type (^WT^*BRAF*) metastatic disease.^19^ In contrary, BRAF mutations that occur outside the codon 600 define a clinically distinct phenotype of metastatic CRC with excellent prognosis.^20^ In a recent study, we demonstrated that the double mutation of APC and PIK3CA in tissue samples from liver metastases was associated with reduced RFS and OS rates, independently of Ras mutation status and surgical management.^21^ However, these features do not correlate well with the inflammatory response of the host to the tumor. As such, analysis of inflammatory signaling can be complimentary to traditional molecular testing.

The mechanism of miR-21, IL-6, and IL-8-induced CRC progression is not clearly understood. Studies performed in preclinical models showed that miR-21 downregulates tumor-suppressor genes including PTEN, and causes upregulation oncologic pathways such us RhoB.^22–27^ In parallel, activation of NF-kB/IL-6 paracrine axis promotes proliferation of CRC epithelial cells,^28,29^ whereas the IL-6-STAT3-mir-34a signaling pathway enhances the EMT phenotype.^30^ In agreement with the above findings, the Src/NF-kB/IL6 axis has been implicated in epithelial transformation in a positive feedback loop.^31^ Similarly, the IL-8-CXCR2 autocrine/paracrine axis has been implicated in proliferation of epithelial cells, EMT-phenotype, and VEGF-independent angiogenesis.^32^ Furthermore, it was proved, in a lung cancer model, that miR-21 can function as ligand to human Toll-like receptor (TLR) family, murine TLR8 in immune cells, triggering a TLR-mediated pro-metastatic inflammatory response including the secretion of cytokines such as TNF-α and IL-6, that ultimately may lead to tumor growth and metastasis.^33^

Poor outcomes and driving processes including EMT, stemness, and angiogenesis have been recapitulated in comprehensive gene expression studies such as the consensus molecular subtypes (CMS). The association of distinct CMSs with tumor inflammation and systemic inflammatory response is currently under evaluation. An initial report demonstrated that out of the four CMSs, two highly express immune-specific gene signatures. The good-prognosis microsatellite unstable (CMS1) is characterized by overexpression of genes specific to cytotoxic lymphocytes. In contrast, the poor-prognosis mesenchymal subgroup (CMS4) expresses markers of lymphocytes and of cells of monocytic origin.^34^ Based on these results, an inflammatory signature might be valuable in distinguishing CMSs and tailor therapy in patients with metastatic CRC.

MicroRNA may have a role in explaining groups defined by comprehensive gene expression in CRC. Network approaches combining transcription factor, methylation, and miRNA analysis demonstrate that up to 74.8% of variation seen in gene expression in mesenchymal vs. other subtypes is explained by miRNAs.^35^ Similarly, an alternate analysis comparing the most differentially expressed miRNAs to each CRC subtype demonstrated that these miRNAs also defined the mesenchymal phenotype.^36^ Both studies also found that downregulation of miR-200 (and family members) contributed significantly to regulating EMT, matrix remodeling, and TNF signaling via NF-kB. A master regulator function for miRNA is suggested by these findings and supports inclusion of miRNAs in future prognostic scores.

While this study demonstrates the potential applications of circulating inflammatory markers to determine aggressiveness of metastatic tumors and responses to treatment, it must be interpreted within the contexts of its limitations. Due to the methodology used, it lacks the external validity of cytokine assays owing to use of percentile cut-offs. Also, this study lacks the direct correlation of circulating markers with expression of the same markers in tissue samples from matched cases. Our assessment of inflammatory markers and clinical characteristics was blinded and samples were prospectively collected, both features that add to the validity of the experimental design but do not obviate the need for direct association of circulating markers with tissue marker expression.

In summary, we demonstrated that a three-marker circulating inflammation signature could be feasible and predictive of outcomes with standard systemic therapy and overall survival, and more importantly demonstrate the potential as a noninvasive assessment of the inflammatory state of the tumor.

## Supplementary information

additional information

Supplementary table 2

Supplementary Table 1

Figure legends
