# Supplementary material for: Circulating inflammation signature predicts overall survival and relapse-free survival in metastatic colorectal cancer
Source: Br J Cancer. 2019 Jan 14;120(3):340–5. doi: 10.1038/s41416-018-0360-y (PMC6353894; doi:10.1038/s41416-018-0360-y)
Supplement: Supplementary file 1 — additional information [file 41416_2018_360_MOESM1_ESM.docx]

**Additional Information:**

**Ethics approval and consent to participate:**

The study was approved by Institutional Review Board (IRB) at the MD Anderson Cancer Center (MDACC). The study was performed in line with the Declaration of Helsinki.

**Consent for publication:**

Human plasma samples for CISIG analysis were obtained after informed consent had been obtained from the patient. The study does not contain any individual person’s data in any form.

**Availability of data and material:**

Data are not available at submission. Cytokine array data will be available upon request to the corresponding author.

**Conflict of Interest:**

The authors declare no conflict of interest

**Funding:**

Work in Dr. Calin’s laboratory is supported by National Institutes of Health (NIH/NCATS) grant UH3TR00943-01 through the NIH Common Fund, Office of Strategic Coordination (OSC), the NIH/NCI grant 1 R01 CA182905-01, a U54 grant – UPR/MDACC Partnership for Excellence in Cancer Research 2016 Pilot Project, a Team DOD (CA160445P1) grant, a Ladies Leukemia League grant, a CLL Moonshot Flagship project, a SINF 2017 grant, and the Estate of C. G. Johnson, Jr. Work in Dr. Kopetz’s laboratory is supported by NIH grants R01 CA184843, R01 CA172670, R01 CA187238, and a CRC Moonshot project.

**Authors’ contributions:**

| Author (initials) | Design of the study | Data collection | Data interpretation | Statistical analysis | Write manuscript |
| --- | --- | --- | --- | --- | --- |
| AV | x |  | x | x | x |
| AK | x | x |  |  | x |
| JD |  |  | x | x | x |
| NS |  | x | x |  |  |
| ML |  | x | x |  |  |
| RLF |  |  | x | x |  |
| CI |  | x | x |  |  |
| MS |  | x | x |  |  |
| JM |  | x | x |  |  |
| DM | x | x | x |  |  |
| MO | x | x |  |  |  |
| HT | x | x |  |  |  |
| JH | x | x | x |  |  |
| YSC |  | x | x |  |  |
| JNV | x | x | x |  |  |
| GC | x | x | x |  |  |
| SK | x |  | x |  | x |

**Acknowledgements:**

Not applicable
