# Supplementary material for: Circulating inflammation signature predicts overall survival and relapse-free survival in metastatic colorectal cancer
Source: Br J Cancer. 2019 Jan 14;120(3):340–5. doi: 10.1038/s41416-018-0360-y (PMC6353894; doi:10.1038/s41416-018-0360-y)
Supplement: Supplementary file 2 — Supplementary table 2 [file 41416_2018_360_MOESM2_ESM.docx]

**Supplementary Table 2:** Correlation of CISIG with variables that were previously associated with poor prognosis in mCRC.

|  | High CISIG (=1) | Low CISIG (=0) | unadjusted p value | FDR adjusted p value (rounded for sig figs) |
| --- | --- | --- | --- | --- |
|  | 54 | 66 |  |  |
| Age >60, n (%) | 20 (37.0) | 32 (48.5) | 0.21 | 0.30 |
| Female Sex | 25 (46.3) | 28 (42.4) | 0.67 | 0.73 |
| Caucasian Race | 40 (74.1) | 53 (80.3) | 0.42 | 0.55 |
|  |  |  |  |  |
| Left sided tumor | 33 (61.1) | 39 (60.0) | 0.90 | 0.90 |
| Poorly differentiated tumor | 15 (31.9) | 17 (27.4) | 0.61 | 0.72 |
| **Chemotherapy received** | **32 (59.3)** | **25 (37.9)** | **0.02** | **0.04** |
| **Albumin <3.5** | **19 (35.2)** | **1 (1.6)** | **<.0001** | **0.0003 (<0.01)** |
| Bilirubin >0.5 | 27 (50.9) | 23 (35.4) | 0.09 | 0.15 |
| **CEA, mean (SD)** | **571.0 (1144.7)** | **103.6 (351.9)** | **0.006** | **0.01** |
| **Hemoglobin <12.4** | **40 (74.1)** | **27 (41.5)** | **0.0004** | **0.001 (<0.01)** |
| **NLR >5** | **28 (51.9)** | **10 (15.6)** | **<.0001** | **0.0003 (<0.01)** |
| **Platelets >310** | **45 (83.3)** | **25 (38.5)** | **<.0001** | **0.0003 (<0.01)** |
| **WBC >8** | **42 (77.8)** | **21 (32.3)** | **<.0001** | **0.0003 (<0.01)** |

|  | OR (95% CI) | p value |
| --- | --- | --- |
| WBC > 8 | 3.8 (1.4, 10.9) | 0.01 |
| Albumin < 3.0 | 19.0 (2.2, 164.1) | 0.01 |
| Platelets > 310 | 3.9 (1.3, 11.9) | 0.02 |
| Hemoglobin < 12.4 | 2.8 (1.02, 7.7) | 0.046 |
