# Supplementary material for: Circulating inflammation signature predicts overall survival and relapse-free survival in metastatic colorectal cancer
Source: Br J Cancer. 2019 Jan 14;120(3):340–5. doi: 10.1038/s41416-018-0360-y (PMC6353894; doi:10.1038/s41416-018-0360-y)
Supplement: Supplementary file 3 — Supplementary Table 1 [file 41416_2018_360_MOESM3_ESM.docx]

**Supplementary Table 1: The correlation between inflammatory markers and relapse free survival after hepatectomy.**

|  | **Median RFS**  **High (m)** | **Median RFS Low (m)** | **HR** | **95% CI** |
| --- | --- | --- | --- | --- |
| **Resectable cohort** | | | | |
| MiR-21 | 19.7 | 24.2 | 1.434 | 0.788-2.611 |
| IL-6 | 18.4 | 28.7 | 1.359 | 0.800-2.307 |
| IL-8 | 21.7 | 24.2 | 1.225 | 0.721-2.801 |
| Inflammatory signature | 18.4 | 31.4 | 2.092 | 1.195-3.662 |
