# Supplementary material for: Circulating inflammation signature predicts overall survival and relapse-free survival in metastatic colorectal cancer
Source: Br J Cancer. 2019 Jan 14;120(3):340–5. doi: 10.1038/s41416-018-0360-y (PMC6353894; doi:10.1038/s41416-018-0360-y)
Supplement: Supplementary file 4 — Figure legends [file 41416_2018_360_MOESM4_ESM.docx]

**Figure legends:**

**Figure 1: Relapse free survival in patients with liver-metastatic CRC after hepatectomy.** Relapse free survival as determined by the time between hepatectomy and relapse of the disease in patients with; A) high vs low IL-6, B) high vs low miR-21, C) high vs low IL-8, and D) positive vs negative Inflammatory signature.

**Figure 2: Overall survival in patients with liver-metastatic CRC that underwent hepatectomy.** Overall survival in patients with; A) high vs low IL-6, B) high vs low miR-21, C) high vs low IL-8, and D) positive vs negative Inflammatory signature.

**Figure 3: Overall survival in patients with liver-metastatic CRC that were not candidates for hepatectomy.** Overall survival in patients with; A) high vs low IL-6, B) high vs low miR-21, C) high vs low IL-8, and D) positive vs negative Inflammatory signature.

**Table 1: Patient Characteristics**

**Table 2: The prognostic value of circulating inflammatory markers and the inflammatory signature in metastatic CRC.**

**Table 3: Cox Proportional Hazards Model.** Inflammatory signature is an independent predictive marker of survival in patients with unresectable disease.

**Supplementary Table 1:** The correlation between inflammatory markers and relapse free survival after hepatectomy.

**Supplementary Table 2:** Correlation of CISIG with variables that were previously associated with poor prognosis in mCRC.
